# First evidence of hepatitis E virus infection in a small mammal (yellow-necked mouse) from Croatia

**DOI:** 10.1371/journal.pone.0225583

**Published:** 2019-11-21

**Authors:** Jelena Prpić, Tomislav Keros, Marko Vucelja, Linda Bjedov, Oktavija Đaković Rode, Josip Margaletić, Boris Habrun, Lorena Jemeršić

**Affiliations:** 1 Croatian Veterinary Institute, Zagreb, Croatia; 2 Forestry Faculty University of Zagreb, Zagreb, Croatia; 3 University Hospital for Infectious Diseases “Dr. Fran Mihaljević”, Zagreb, Croatia; 4 University of Zagreb School of Dental Medicine, Zagreb, Croatia; CEA, FRANCE

## Abstract

Since the role of wild rodents/small mammals in hepatitis E virus (HEV) epidemiology has been a subject of considerable debate, this study was conducted to investigate the potential presence of HEV RNA in small rodents collected within their natural habitats and to detect if they can be potential reservoirs of the virus. A total of 483 small rodents were captured using snap traps placed at 11 regions in Croatia. Sampling was undertaken in 2008 and repeated from 2010 to 2014. Liver samples were tested for the presence of HEV RNA. HEV RNA was detected in only one liver sample (0.21%) originated from *Apodemus flavicollis* from the location Medvednica, nearby Zagreb collected in 2014. According to the sequence analysis, the isolate has shown to be a member of *Orthohepevirus A* species, genotype HEV-3. The genotyping results confirmed grouping into subtype 3a, general cluster 3abchij.The detected HEV strain showed to be genetically highly related to strains found in humans and/or domestic pigs and wild boars from Croatia. Our finding indicates that wild small mammals could play a role in the epidemiology of HEV-3 infection and therefore should be taken under consideration as potential reservoirs or/and transmitters of the disease. However, further investigation is needed to recognize their potential for maintaining the infection in natural conditions.

## Introduction

Hepatitis E virus (HEV) is classified as a member of the family *Hepeviridae*, genus *Orthohepevirus*[1, 2]. The genus includes four genotypes: *Orthohepevirus A*, *B*, *C* and *D*. *Orthohepeviru*s*A* have been isolated from humans, swine (pigs and wild boars), deer, mongooses, rabbits and camels[1, 2]. *Orthohepevirus B* have been isolated from birds, *Orthohepevirus C* from rats, ferrets and minks[1, 2]. *Orthohepevirus D* includes strains from bats [1, 2]. The largest species, *Orthohepevirus A*, comprises seven genotypes [1, 2]. Genotypes 1 to 4 HEV (HEV-1 to -4) of *Orthohepevirus A* are known to cause disease in humans. HEV-1 and HEV-2 infect only humans, causing epidemics in developing countries, while HEV-3 and HEV-4 are zoonotic and can spread from animals to humans [3, 4]. The zoonotic potential of other *Orthohepevirus* genotypes (B, C, D) derived from various animals remains unclear.

HEV-3 is native and widespread throughout Europe [5], including Croatia [6]. Active infections and high seroprevalence are measured on Croatian pig farms (up to 92%), in wild boar (up to 30%) as well as in humans (up to 15%) [7, 8, 9]. In contrast to other hepatitis viruses, experimental models other than nonhuman primates have been established, such as pigs, sheep and laboratory rats [9, 10]. Identification of anti-HEV antibodies in domestic animals like pigs, sheep and cattle [11], as well as the isolation and characterization of HEV isolates from domestic pigs similar to human HEV [12, 13] confirm that animals can be reservoirs for HEV.

A high prevalence of seropositive rodents in the USA and Asia[14–18] provides strong evidence that rodents could be potential reservoirs of HEV [14, 16, 17]. Recently, in Europe and Asia, in several small rodent and insectivore species HEV RNA *Orthohepevirus* C genotype was also detected [19–22]. However, studies conducted on rodents, other than rats, are lacking, so the evaluation of rodents as competent reservoirs of HEV-3 still remains uncertain even though HEV-1 and HEV-3 have experimentally been derived from human liver chimeric mice [23]. Moreover, a successful infection of *Mus musculus* mouse liver cell culture with HEV-3, supports the hypothesis that rodents may be competent hosts [24].

Continental Europe, including Croatia, has a great wildlife biodiversity, including a wide range of rodent species and other small mammals that have been reported as reservoirs of several clinically important bacterial, protozoan, and viral diseases [17]. Due to its diverse natural habitats and possible wildlife/domestic animals/public interactions, there is a potential for many rodent-borne zoonoses. This study presents the first report of a natural HEV-3 infection of a small rodent.

## Materials and methods

### Sample collection

**Fig 1:**
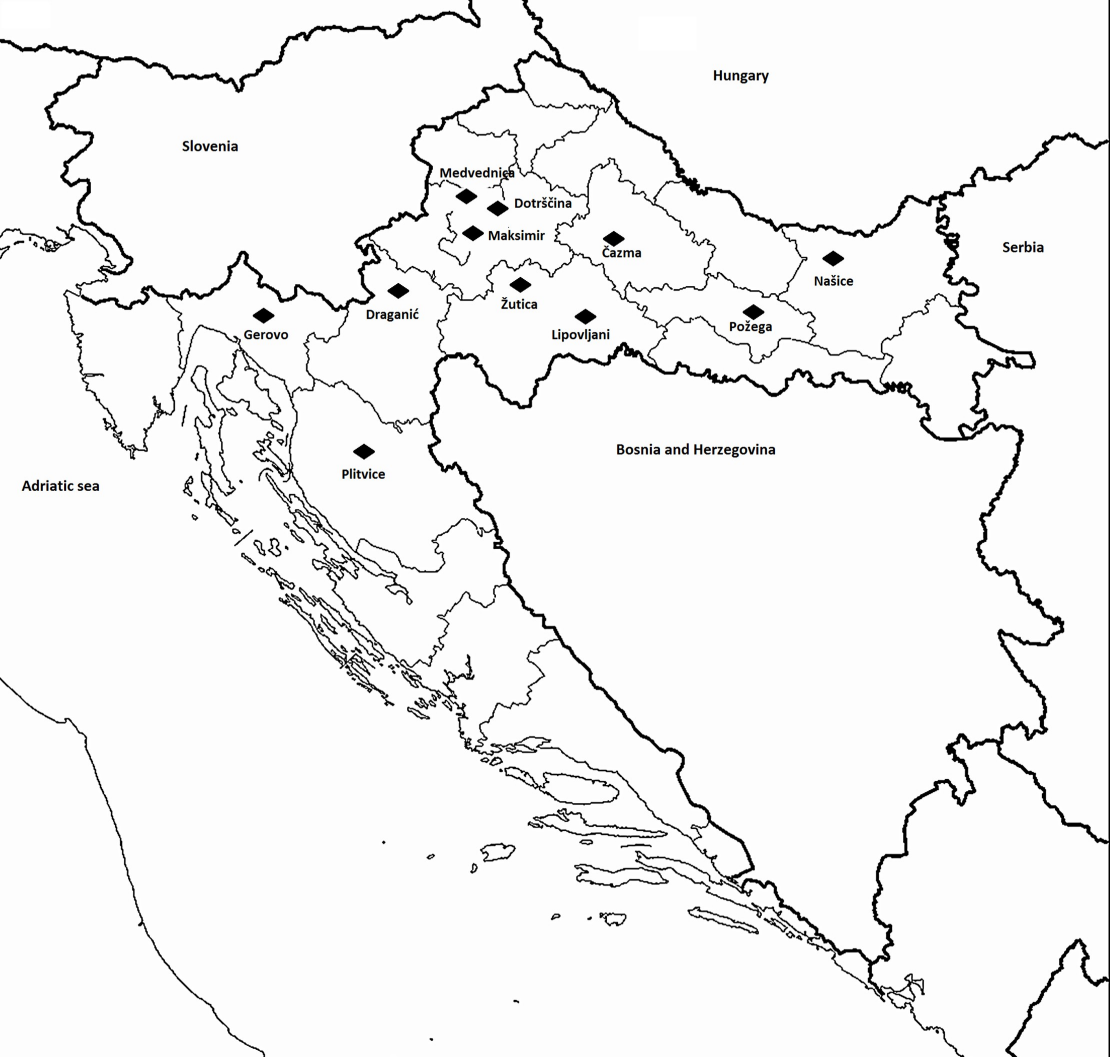
Map of Croatia indicating the regions where the small rodents were trapped.

A total of 483 small rodents (159 *Myodesglareolus*, 3 *Apodemussylvaticus*, 44 *Apodemusagrarius*, 242 *Apodemusflavicollis*, 17 *Apodemusspp*, 14 *Microtus agrestis* and 4 *Microtus arvalis*) were captured using snap traps (Mini T-rex Mouse Snap Trap, Bell Laboratories inc.) placed at 11 ecologically diverse mountainous and lowland localities (Medvednica, Gerovo, Plitvice; Maksimir, Dotrščina, Lipovljani, Čazma, Žutica, Draganić, Našice and Požega) in Croatia (Fig 1). Trapping was performed using linear transect method. Sampling was done in 2008 and in the time period from 2010 to 2014 as part of the projects supported by Ministry of Science, Education and Sports (068-1430115-2119, „The forest ecoystems as natural foci of the hantaviruses and leptospira”) and by Croatian Science Foundation (IP-11-2013-4250, “The role of biotic agents on vitality of narrow-leafed ash (*Fraxinus angustifolia* Vahl.) in Croatian floodplain forests“), designed to assess the distribution of small rodents in diverse forest ecosystems in Croatia [25]. The animals were labelled, weighted, measured, and then aseptically dissected. All rodents were morphologically characterized. Animal experimentation guidelines of the American Society of Mammologists were followed during all animal work [26]. Sampling was done in accordance with good veterinary practice that is fully complied with the Croatian Animal Protection Act (Official Gazette of the Republic of Croatia, No. 102/2017). Croatian legislation is harmonized with the Code of good veterinary practice. (https://ec.europa.eu/digital-agenda/en/content/federation-veterinarians-europe-code-good-veterinary-practice)(http://www.fve.org/news/publications/pdf/gvp.pdf). The permits for field sampling were in agreement and oversight of the Croatian forests Ltd., Public Institution "National park Plitvice lakes" and Public Institution "Nature park Medvednica". This study did not include any experiments or trials. Collected liver samples were stored at -80°C until further investigation.

### Detection of viral RNA

Total RNA was extracted from liver homogenate using MagMAX Pathogen RNA/DNA Kit (Thermo Fisher Scientific, USA) according to the manufacturer's instruction. Tissue samples (100mg of each) were homogenized by using the FastPrep-24 machine, model 6004–500 (MP Biomedicals, Santa Ana, California, USA) and lysing matrix A, D, and for tonsils matrix M, with addition of 1ml sterile phosphate buffered saline (PBS). The supernatants were decanted into sterile test tubes and stored at -80°C until further testing. For positive animals additional organ samples were tested (lungs, kidney, spleen, colon, heart and heart transudate). To identify HEV RNA carriers, an one-step quantitative real time RT-PCR protocol [27] for detecting highly conserved fragment within ORF3 (open reading frame 3) was carried out. All positive samples (samples containing HEV RNA detectable in a cycle threshold value lower than 40) were re-tested by a nested RT-PCR protocol [28] for detection of the variable fragment within ORF1. The amplification procedures for ORF1 and ORF3 regions are described by Jemeršić et al. [6]. PCR products were separated by agarose gel electrophoresis in 1.5% agarose gel stained with a fluorescent dye-GelStar (Lonza, Belgium) and visualized by UV transillumination. For positive control sequences of previously detected fragments of HEV RNA genotype 3 were used (derived from positive swine sera submitted to GenBank under accession no. KT583116, grouped into HEV3c subtype, with Ct-value 29). Negative controls were aliquots of ultrapure water.

### Sequence analysis

PCR product was purified (Wizard SV Gel and PCR Clean-Up system (Promega, USA) and sequenced by Macrogen Inc. (Amsterdam, Netherlands). The sequence of 242 base pairs was submitted to GenBank (accession no. MK456392). Phylogenetic grouping and clustering were based on comparison with sequences retrieved from the GenBank, using algorithm BLAST (http://www.ncbi.nlm.nih.gov). Sequences were aligned with ClustalW 1.6 and analyzed using MEGA7 [29], based on published recommendations [2]whereas the trees were generated using the neighbour-joining method applying the Kimura 2-parameter evolutionary model.

## Results and discussion

**Fig 2:**
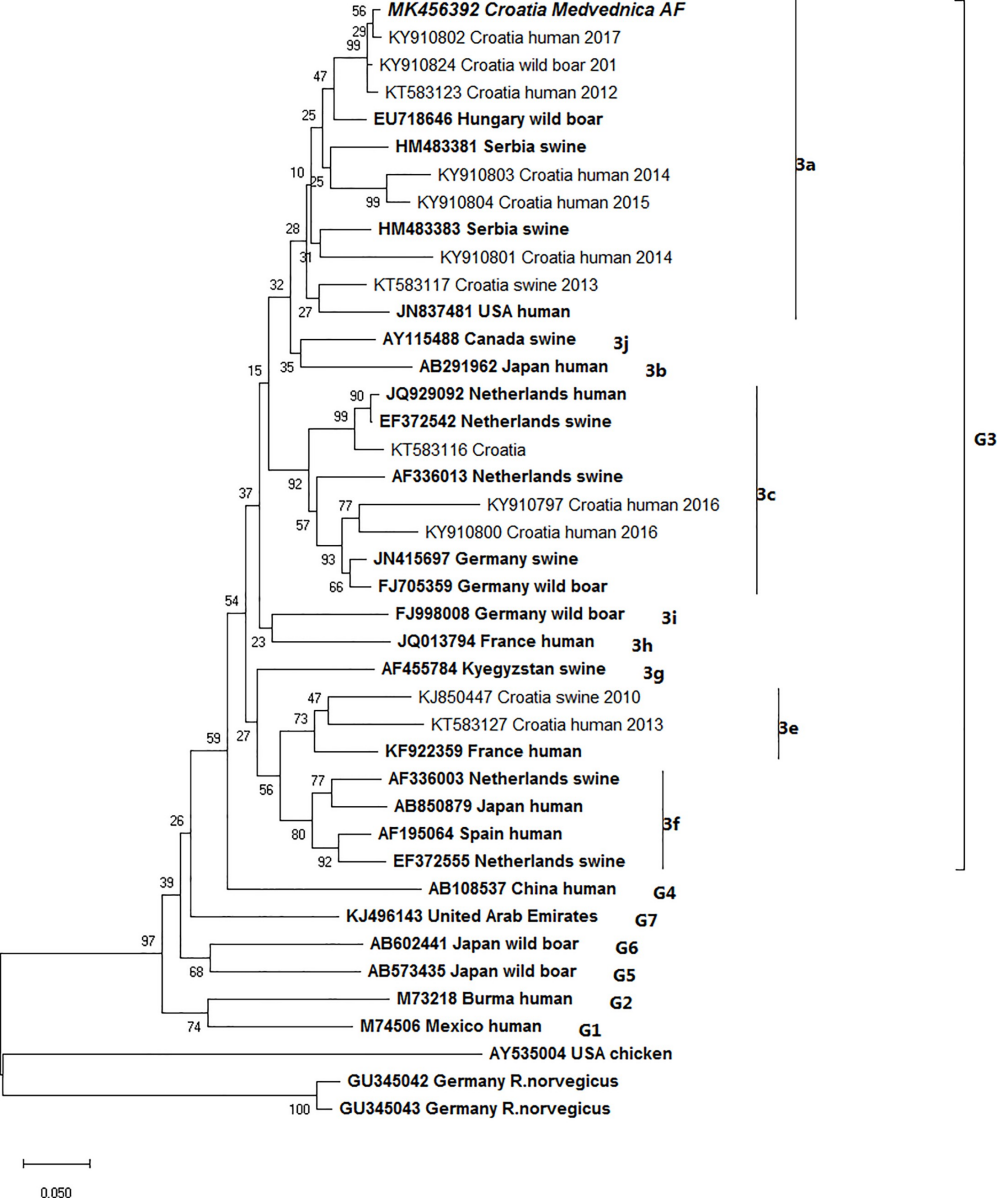
Neighbour-joining phylogenetic tree obtained by the analysis of the partial 242nt sequence of the ORF1 region of HEV-3 isolate derived from yellow-necked mouse sample from Croatia (MK456392). Genetic distances were calculated using the Kimura two-parameter method. Bootstrap values calculated from 1000 replicates are presented next to the tree nodes. The bar represents 0.05 nucleotide substitutions per site.

Viral RNA was detected in only one liver sample (0.21%, 1/483) derived from *A*. *flavicollis* (yellow-necked mouse) from Medvednica mountain (Zagreb County). The mouse was captured in 2014, near a mountain cottage, a favourite hiking site. Ct-value of the positive rodent liver sample was 31. Other tissues and organs of the positive animal were negative for HEV. The phylogenetic analysis of the partial 242ntORF1 obtained sequence (Fig 2) showed that the HEV sequence derived from *A*. *flavicollis* grouped into genotype 3 of *Orthohepevirus* A species, subtype 3a (clade 3abchij) defined by Smith et al. [2]. The obtained sequence from Medvednica is strongly genetically related to other Croatian sequences, as those derived from humans in 2012 (KT583123, Zagreb county, 98.38%), 2014 (KY910801, Zagreb county, 86.58%; KY910803, Zagreb county, 90.65%) and 2017 (KY910802, Dubrovnik-Neretva county, 98.78%), wild boar from 2016 (KY910824,Vukovar-Srijem county, 98.78%) and a domestic swine from 2013 (KT583117, Međimurje County, 92.27%). The predominant strains in Croatia belong to subtypes 3a and 3c and thus, can be considered endemic [6].

Rodents and small mammals are hosts and vectors of a wide range of zoonotic pathogens. Rodent-borne agents can be transmitted to humans directly, mostly through rodent bites and indirectly by contact with their contaminated excretions or by arthropod vectors including ticks, fleas, and mites. Few studies showed simultaneous multiple infections in rodents and small mammals in Croatia and Austria [30, 31, 32]. Seroprevalence studies done in the United States and Asia in the 1990s identified many rat species positive for HEV antibodies [14–18], but the role of wild rodents and small mammals as reservoirs in HEV epidemiology is still unclear [33]. Whether HEV-3 can be added to the rodent-borne pathogens list awaits further research. Low genetic diversity of HEV-positive samples detected across the United States [33], suggest that only a limited number of HEV genotype 3 strains may be capable of infecting *Rattus* spp. rats and other rodents (i.e., *Mus* spp.). Since there are no published reports of investigation for the presence of HEV-3 in rodents and small mammals in natural conditions in general, this report brings new knowledge on the HEV-3 host range and its zoonotic potential.

The detection of hepatitis E infections is arising in Croatia, with the occasional appearance of sporadic infections in humans. A high prevalence of HEV RNA and anti-HEV antibodies in swine and wild boars has also been observed. Therefore the detection of a naturally infected forest mouse as a competent host of a HEV-3 genotype that has been previously derived from humans, swine and wild boars may present an epidemiological interspecies ‘‘link” between wildlife and domestic swine and have an important role in obtaining the virus in the environment.

The HEV-3 positive mouse was captured nearby the Croatian capital, the city of Zagrebon the mountain Medvednica (Zagreb County, Nature Park Medvednica), on a location that is very attractive for hikers, cyclists, and tourists. Interestingly, the majority of HEV-3 infected patients in Croatia originated from Zagreb County[6]. Previous studies showed that HEV-3 is circulating in humans from 2012 [34, 35] and among swine and wild boars in Croatia since 2009 [7, 36]. Moreover, the majority of swine farms and the highest density of wild boars are located in Northern Croatia [6]. The detected high similarity of here obtained HEV-3 mouse isolate and previously obtained Croatian human, wild boar and swine isolates [6, 7] indicates possible interspecies transmission of HEV and/or existence of an accessible mutual source of infection.

Even though HEV has primarily hepatic tropism, it can be found relatively active in other tissues when extrahepatic post-infection manifestations have been recorded, especially in immunocompromised subjects[37, 38]. Data on HEV tissue tropism are still scarce therefore we tested all other accessible tissues and organs (lungs, kidney, spleen, colon, heart and heart transudate) of the positive mouse. Only the liver tissue was HEV-3 RNA positive in our *A*. *flavicollis*, while other tested tissues remained negative. Since HEV infection in naturally infected animal reservoirs, such as domestic swine, show no signs of disease [39, 40], an asymptomatic, non-persistent HEV infection in yellow-necked mice, with reduced HEV detection in other tissues apart from the liver, is possible.

The HEV infection in the yellow-necked mouse occurred chronologically later than in swine and wild boars since all samples collected from rodents from the earlier periods shown to be negative indicating that our *A*. *flavicollis* is a more recent HEV host and/or that it was recently infected and that the virus remains in the mouse for a shorter period. According to Maneerat et al.[41] active virus propagation with detectable HEV RNA in sera and feces of rats is possible early post-infection (up to 30 days). Furthermore, studies done on rats demonstrated non-persistent infections [42]. Therefore, time limited short viremia could be a reason for detecting only one positive *A*. *flavicollis* indicating that the percentage of Croatian rodents carrying HEV-3 could be higher. Unfortunately, due to a lack of sera samples we cannot foresee the real picture of rodent HEV-3 seroprevalence in Croatia.

This study showed that some species of small mammals should be further investigated to evaluate their role as an epidemiological ‘link’ within the wildlife/domestic animal cycle and even a potential public health risk and reservoir of HEV-3 since the strain has been found to be genetically identical to previously detected strains derived from humans, wild boars, and swine. To confirm the true role of rodents as reservoirs or vectors of HEV-3, prolonged surveillance of HEV-3 in rodents and small mammals in Croatia and elsewhere is needed. However, our finding of the first naturally infected small rodent definitely opens a new window within the epidemiology and environmental sustainability of HEV.

## Conclusions

Herein we presented the first detection of a naturally HEV infected small rodent, a yellow-necked mouse, and a report of HEV-3 occurrence and prevalence (0.21%) in wild small rodents from 11 localities in continental mountainous and lowland Croatia. Detected prevalence in Croatian wild small rodents is lower than the prevalence in *Rattu*s spp. rats from the United States (7.85%)[33] and than the prevalence in *Rattu*s *norvegicus* from Germany (6.67%) [43]. The yellow-necked mice can have a role in HEV-3 maintenance and epidemiology, therefore small rodents should be further investigated. Since the derived strain showed to be genetically highly related to strains found in humans and wild and domestic animals from Croatia it indicates that mice can be a link in the viral transmission to other susceptible species, including humans.
